# Evidence for Host Epigenetic Signatures Arising From Arbovirus Infections: A Systematic Review

**DOI:** 10.3389/fimmu.2019.01207

**Published:** 2019-05-31

**Authors:** Gabriella Pequeno Costa Gomes de Aguiar, Claudio Manuel Gonçalves da Silva Leite, Beatriz Dias, Silvania Maria Mendes Vasconcelos, Renata Amaral de Moraes, Maria Elisabete Amaral de Moraes, Antonio Carlos Rosario Vallinoto, Danielle Silveira Macedo, Luciano Pamplona de Goes Cavalcanti, Fabio Miyajima

**Affiliations:** ^1^Postgraduate Programme in Medical and Surgical Sciences, Faculty of Medicine, Federal University of Ceará, Fortaleza, Brazil; ^2^Postgraduate Programme in Pharmacology, Faculty of Medicine, Federal University of Ceará, Fortaleza, Brazil; ^3^Faculty of Medicine, Unichristus University Center, Fortaleza, Brazil; ^4^Center for Drug Research and Development (NPDM), Faculty of Medicine, Federal University of Ceara, Fortaleza, Brazil; ^5^Sao Jose Hospital of Infectious Diseases, Fortaleza, Brazil; ^6^Laboratory of Virology, Institute of Biological Sciences, Federal University of Pará, Belém, Brazil; ^7^Department of Community Health, Faculty of Medicine, Federal University of Ceara, Fortaleza, Brazil; ^8^Institute of Translational Medicine, University of Liverpool, Liverpool, United Kingdom; ^9^Postgraduate Programme in Medical Sciences, Faculty of Medicine, Federal University of Ceará, Fortaleza, Brazil; ^10^Oswaldo Cruz Foundation (Fiocruz), Branch Ceara, Eusebio, Brazil

**Keywords:** systematic review, host epigenetics, microRNAs, histone modifications, arbovirus

## Abstract

**Background:** Arbovirus infections have steadily become a major pandemic threat. This study aimed at investigating the existence of host epigenetic markers arising from the principal arboviruses infections impacting on human health. We set to systematically review all published evidence describing any epigenetic modifications associated with infections from arboviruses, including, but not limited to, microRNAs, DNA methylation, and histone modifications.

**Methods:** A comprehensive search was conducted using the electronic databases PubMed, Science Direct and Cochrane Library from inception to January 4th, 2018. We included reports describing original *in vivo* or *in vitro* studies investigating epigenetic changes related to arbovirus infections in either clinical subjects or human cell lines. Studies investigating epigenetic modifications related to the virus or the arthropod vector were excluded. A narrative synthesis of the findings was conducted, contextualizing comparative evidence from *in vitro* and *in vivo* studies.

**Results:** A total of 853 unique references were identified and screened by two independent researchers. Thirty-two studies met the inclusion criteria and were reviewed. The evidence was centered mainly on microRNA and DNA methylation signatures implicated with secondary Dengue fever. Evidence for recent epidemic threats, such as the infections by Zika or Chikungunya viruses is still scant.

**Conclusions:** Major epigenetic alterations found on arboviruses infections were miR-146, miR-30e and the Dicer complex. However, existing studies frequently tested distinct hypotheses resulting in a heterogeneity of methodological approaches. Whilst epigenetic signatures associated with arbovirus infections have been reported, existing studies have largely focused on a small number of diseases, particularly dengue. Validation of epigenetic signatures have an untapped potential, but concerted investigations are certainly required to deliver robust candidates of clinical utility for diagnosis, staging and prognosis of specific arboviral diseases.

## Introduction

Arboviruses are virus transmitted to humans by arthropod vectors (1). They are grouped into five families: *Flaviviridae* (e.g., Dengue and Zika viruses), *Togaviridae* (e.g., Chikungunya virus), and the less commonly reported *Bunyaviridae, Reoviridae* and *Rhabdoviridae*.

The global distribution of arboviruses is directly linked to the areas cohabited by its vectors and most of them typically occur amongst tropical and subtropical regions (2, 3). For instance, Brazil has recently been plagued by consecutive epidemic seasons, initially with Dengue, then Zika and more recently Chikungunya, during which over 300,000 cases were reported nationwide. (1, 4–6). Ingeniously, infections of arthropod vectors do not seem to affect their fitness and are typically non-pathogenic and lifelong. By contrast, infections of the human host with these viruses, particularly *Flaviviridae* and *Togaviridae* families, are generally pathogenic, often acute and causing viremia (7). This combination largely contributes to their successful viral spread and persistence, accounting for considerable medical and economic burden to healthcare systems of endemic nations (3).

Although arboviruses undergo constant genetic evolution as they typically lack polymerases with proofreading activity and thus exhibit significant mutation frequencies, there is also strong purifying selection resulting from the requirement for their replication in two disparate hosts, and from a balanced fitness trade off they have deployed intricate mechanisms of interaction with their alternate hosts (8). The identification of such interactions may provide insights into the mechanistic basis of the infections with advanced investigative strategies aiming to tackle the current lack of solutions for intervention and therapy.

The role of environmental stimuli, such as a trauma or infection, have always been assumed but largely unexplored until recently due to the unavailability of experimental approaches. Epigenetics is a promising research field and represents a shift in paradigm as it takes into account environmental stimuli modifying gene regulation without necessarily changing the host's core DNA sequences.

In general, the most common epigenetic modifications include changes in chromatin packaging through reversible post-translational modifications in histones (e.g., acetylation, deacetylation), as well as changes in gene regulatory positions, such as gene promoter or gene enhancers, through reversible methylation/acetylation modifications of the DNA nucleotides (9). Of note, the downstream impact of epigenetic alterations may be life long, persisting through subsequent generations of cells during replication (also known as transgenerational gene regulation). Furthermore, other epigenetic events have been proposed that includes the selective neutralization of the messenger RNAs (mRNAs) used in protein translation by interference RNA (iRNA), such as micro RNA (miRNA), and small interfering RNA (siRNA) (10).

The pathogenicity and severity of clinical manifestations posed by arbovirus are likely to involve epigenetic regulation mechanisms, present at the host-pathogen interface. One example is iRNA as a host mechanism acting as an immune response against exogenous molecules, including viruses. In response, viruses are capable of neutralizing host cell defenses by producing factors known as iRNA suppressors (11).

Epigenetic changes can be triggered by several extrinsic factors and are relevant for the host homeostasis and bodily functions, including the control of diseases, which in turn can elicit specific molecular signatures. Thus, the evaluation of epigenetic changes arising from specific arbovirus infections and connected to the immune activation can provide insights into pathophysiological mechanisms prompting the course and severity of a given arbovirus condition.

To the best of our knowledge, no previous review has synthesized the host epigenetic signatures of arbovirus infections. Thus, we conducted a systematic review of original *in vivo* and *in vitro* studies investigating human epigenetic markers arising from infections with arboviruses in both clinical subjects and human cell lines.

## Methods

### Search Strategy

This systematic review followed an *a priori* defined yet unpublished protocol. The electronic databases PubMed/MEDLINE, Science Direct and Cochrane Library were searched from inception to January 4^th^, 2018. The detailed search string used in this systematic review is provided in the supplementary online material that accompanies the online version of this article. We followed the *Preferred Reporting Items for Systematic Reviews and Meta-Analyses* (PRISMA) statement (12).

### Eligibility Criteria

The inclusion criteria included: original studies, *in vitro* or *in vivo*, regarding epigenetic markers related to human infection by arboviruses. *in vitro* or *in vivo* studies comprised either human cell lines, or clinical subjects. Epigenetic markers consisted of changes in DNA methylation, histone post-translational modifications that affected gene expression, and iRNA suppression mechanisms. No language restrictions were applied, and reports from any country were included to avoid publication bias. Non-original publications, such as reviews, letters or comments were excluded. Studies using animal cell lines, pre-clinical animal studies, *in silico* research, as well as investigations limited to epigenetic modifications on the arbovirus or on the arthropod vector were also excluded.

### Study Selection

Two investigators (GA and BD) independently screened the titles/abstracts of the unique references. After this primary screening, the full-texts of selected reports were obtained, and the same authors independently reviewed each article to determine its final inclusion in the review. Whenever a consensus could not be reached, a third author (CL or FM) made the final decision regarding inclusion.

### Data Extraction

Two authors (GA and BD) independently extracted the data from the included references using a standardized form. Discrepancies were resolved through consensus. The following information was recorded for each study: author, year, virus/serotype, disease, epigenetic marker, and main results. For *in vitro* studies, we also noted the investigated cell line. For *in vivo* studies, the clinical characteristics of the sample were recorded.

### Evidence Synthesis

Due to the heterogeneity of study design, participants and outcomes, we conducted a narrative synthesis of the included studies, summarizing the findings with respect to each arbovirus.

## Results

**Figure 1:**
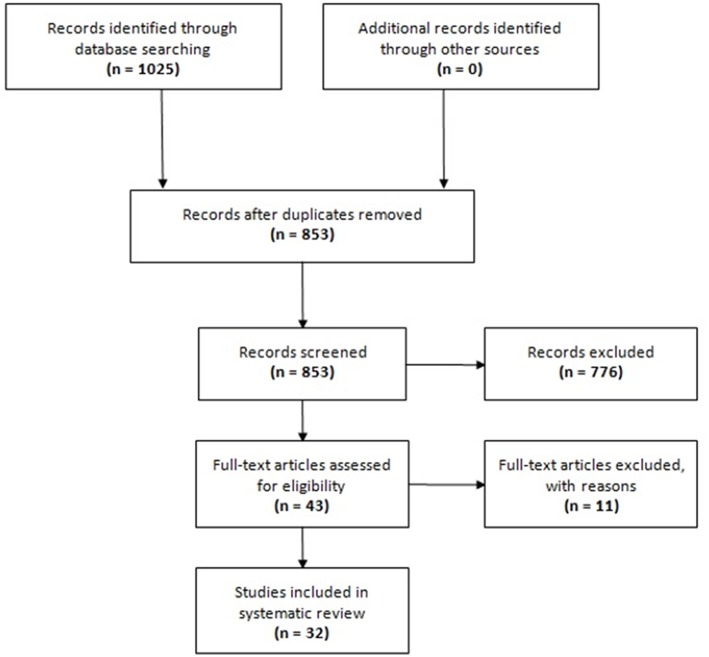
Flow chart of study selection.

The search in electronic databases yielded 1,025 references. No additional references were found from manual searching the reference lists of included articles. After exclusion of duplicates, 853 unique references were selected for title/abstract screening, of which 43 were eligible for full-text review. Eleven full-text articles were excluded with reasons (see Table S1 in the Supplementary Material that accompanies the online version of this article). Finally, 32 articles were included in this systematic review (13–44). The PRISMA flowchart of study selection for this systematic review is provided in Figure 1.

### Characteristics of Included Studies

Overall, this systematic review included 7 *in vivo* (16, 20, 30, 34–37) and 25 *in vitro* studies (13–15, 17–19, 21–29, 31–33, 38–44). The *in vitro* studies used different human cell lines, including three with human monocytes cells lines (22, 23, 42), six with human liver cell lines (13, 15, 19, 24, 31, 40), six studied neuronal cells (26, 28, 32, 38, 41, 44), five used human-derived renal cell lines (15, 17, 25, 27, 33). Two studies were based on human fibroblasts. (14, 21), isolated studies were conducted with natural killer cells (34), epithelial cells (29), human trophoblasts (43), and human vascular endothelial cells (22).

The *in vivo* studies included a total of 171 cases and 102 controls. Sample sizes varied from 6 to 89 participants. All these studies were published in English. Description and characteristics of included studies are provided in Table 1.

**Table 1:** Characteristics and main findings of the studies investigating epigenetic markers associated with arbovirus infections in humans.

**References**	**Virus**	**Study type**	**Epigenetic alteration**	**Results**	**Cell line**
Bayer et al., (43)	ZIKV	*in vitro*	chromosome 19 microRNA cluster overexpression	C19MC miRNAs limit ZIKV infection	human throphoblasts
Bogerd et al. (19)	WNV and DENV	*in vitro*	Dicer mRNA	DENV and WNV do not express any viral miRNAs in human cells and neither DENV nor WNV induce the production of viral siRNAs in infected human somatic cells	Huh7 cells, a type of human liver cell line
Casseb et al. (29)	DENV	*in vitro*	Drosha, DGCR8, and Dicer mRNAs	Drosha, DGCR8, and Dicer mRNAs are down-regulated in human cells infected with DENV-4	epithelial cells
Chen et al. (20)	DENV	*in vivo*	miR-150 overexpression	reduced SOCS1 expression was significantly associated with up-regulated miR-150 levels	peripheral blood mononuclear cells
Colpitts et al. (13)	DENV	*in vitro*	Nuclear histone proteins H2A, H2B, H3 and H4 as target of the capsid protein of the virus	All four core histones were pulled out of the cell lysates by DEN2 C and were identified with high percent coverage and low expectation values	Huh7 cells, a type of human liver cell line
Demir et al. (37)	CCHFV	*in vivo*	downregulation of miR-146a and upregulation of miR-451 and miR-31	downregulation od miR-146aand upregulation of hsa-miR-451 and hsa-miR-31 leads to augmentation of proinflammatory cytokines	peripheral blood mononuclear cells
Devhare et al. (38)	ZIKV	*in vitro*	reduced p53 phosphorylation	ZIKV strains induce human neural stem cells (hNSC) growth inhibition and display distinct E glycoprotein localization	human neural stem cells
Diosa-Toro et al. (39)	DENV	*in vitro*	miR-3614-5p overexpression	miR-3614-5p regulated ADAR1 expression, a protein that facilitates viral replication	primary human macrophage
Escalera-Cueto et al. (24)	DENV	*in vitro*	miRNA Let-7 overexpression	Let-7c has an antiviral effect through the modulation of BACH1 and HO-1	Huh7 cells, a type of human liver cell line
Gomes et al. (30)	DENV	*in vivo*	Methylation of cytokine genes	Significant frequency of demethylation in the region of the TNF-a promoter in DENV infected patients	Total RNA was isolated from whole blood
Kakumani et al. (31)	DENV	*in vitro*	human microRNA mir-126	The chaperone and the miRNA, hsa-mir-126-5p restricts dengue virus replication in human cell lines	Huh7 cells, a type of human liver cell line
Kakumani et al. (15)	DENV	*in vitro*	RNAi suppression	Downregulation of iRNA factors as Dicer, Drosha, Ago1 and Ago2. Also, NS4B, a viral protein, as a RNAi supressor.	Huh 7 and HEK293T (human embryonic kidney cells)
Kakumani et al. (25)	DENV	*in vitro*	RNAi suppression	DENV non-structural protein 3 suppresses human miRNA machinery	human embryonic kidney (HEK) HEK293T cells
Kanokudom et al. (40)	DENV	*in vitro*	microRNA 21 overexpression	miR-21 is significantly increased upon DENV 2 infection and that expression of this miRNA promotes DENV replication	HepG2 cells, a human liver cancer cell line
Kozak et al. (41)	ZIKV	*in vitro*	miR-30e-3p, miR-30e-5p, miR-17-5p	induction of genetic pathway of autophagy including CHOP and GADD34	astrocytes
Kumari et al. (32)	JEV	*in vitro*	miR-34c-5p suppression	miR-34c-5p which is suppressed during JEV infection and overexpression of this miRNA modulates JEV induced proinflammatory cytokine production	human microglial cells
Lichinchi et al. (33)	ZIKV	*In vitro*	human m6A RNA	m6A RNA Methylation Modulates the ZIKV Life Cycle	human HEK293T cells
Liu et al. (34)	DENV	*In vivo*	miR-27a*, miR-30e, and miR-378 were down-regulated	miR-378, but not miR-27a* or miR-30e, suppressed GrzB expression in NK cells	NK cells
Ouyang et al. (35)	DENV	*In vivo*	miR-21-5p, miR-590-5p, miR-188-5p, and miR-152-3p overexpression, whereas miR-146a-5p was down-regulated	miR-21-5p and miR-146a-5p could distinguish dengue-infected patients with preferable sensitivity and specificity	Total RNA was isolated from whole blood
Pham et al. (14)	DENV	*In vitro*	miR-142 incorporation	Incorporation of miR-142 target sites into the 3UTR of DENV-2 confers endogenous attenuation of the virus in a cell-specific manner	human fibroblasts
Pu et al. (42)	DENV	*In vitro*	miR-146a overexpression	miR-146a significantly blocked DENV2-induced autophagy	A549 cells (adenocarcinoma human alveolar basal epithelial cells) and THP-1 cells (a human monocytic cell line)
Qi et al. (16)	DENV	*In vivo*	miR-4290,−4279,−625*, -let-7e,−1290,−33a,−378,−1246,−767-5p,−320c,−720,−491-3p,−3647,−451 and−4286, miR-106b,−20a,−30b and−3653	Upregulated miRNA subsets included: miR-4290,−4279,−625*, -let-7e,−1290,−33a,−378,−1246,−767-5p,−320c,−720,−491-3p,−3647,−451 and−4286.Downregulated miRNA subsets included: miR-106b,−20a,−30b and−3653	peripheral blood mononuclear cells
Rastogi et al. (44)	JEV	*In vitro*	48 differentially expressed microRNAs26/02/2018 34 are significantly up regulated and 14 are down regulated	miR-32-5p is up regulated in JEV infected human microglia with respect its pleiotropic to control. KLF4 (Kruppel-like-factor 4) gene was predicted as one of its target. The down-regulation of miR-432-5p and NEDD4L (neural precursor cell expressed developmentally down-regulated protein 4) has been identified as one of the potential target as well	human microglial cells
Saxena et al. (17)	CHIKV	*In vitro*	miR-744, miR-638, miR-503	miRNA expression profiling revealed regulation of 152 miRNAs post CHIKV infection	HEK293T cells
Selvamani et al. (21)	CHIKV	*In vitro*	miR-146a overexpression	CHIKV infection induced the expression of cellular miR-146a, which resulted into down-regulation of TRAF6, IRAK1, IRAK2 and increased replication of CHIKV in human synovial fibroblasts. Downregulation of TRAF6, IRAK1 and IRAK2 led to downstream decreased NF-kB activation through negative feedback loop	Human Synovial Fibroblasts
Sharma et al. (26)	JEV	*In vitro*	miR146-a overexpression	Exogenous overexpression of miR-146a led to suppression of NF-κB activation and abrogation of Jak-STAT pathway upon JEV infection which led to downregulation of interferon-stimulated genes (IFIT-1 and IFIT-2) and facilitated viral replication	human microglial cells
Slonchak et al. (27)	WNV	*In vitro*	miR-532-5p overexpression	miR-532-5p exhibits antiviral activity against West Nile virus via suppression of host genes SESTD1 and TAB3 required for virus replication	Human embryonic kidney cells (HEK293)
Tambyah et al. (36)	DENV	*In vivo*	Overexpression of a cluster of 12 miRNA (miR-450b-5p,−491-5p,−499a-3p,−512-5p,−615-5p,−624-5p,−892b,−1204,−1225-5p,−3121-3p,−4259 and−4327)	Expression patterns of 12 miRNAs (miR-450b-5p,−491-5p,−499a-3p,−512-5p,−615-5p,−624-5p,−892b,−1204,−1225-5p,−3121-3p,−4259 and−4327) were seen to be significant and specific for acute dengue cases. Expression of miR-24-1-5p, miR-512-5p and miR-4640-3p distinguished mild dengue from those exhibiting liver complications whereas miR-383 was significantly upregulated in mild dengue compared to those diagnosed as severe dengue with fluid accumulation.	Total RNA was isolated from whole blood
Wu et al. (22)	DENV	*In vitro*	miR-223 overexpression	miR-223 overexpression controls DENV-2 replication by negatively regulating microtubule destabilizer STMN1 gene	EAhy926 cells, a human vascular endothelial line
Wu et al. (18)	DENV	*In vitro*	miR-146a overexpression	miR-146a facilitates DENV replication by targeting TRAF6 and consequently reducing IFN-B levels	human monocytic cell THP-1, a model for human monocytes
Zhu et al. (28)	JEV	*In vitro*	MicroRNA-15b overexpression	miR-15b Modulates JEV– mediated Inflammation via Targeting RNF125	human astrocytoma cell and HeLa cells
Zhu et al. (23)	DENV	*In vitro*	induction of MicroRNA-30e*	miR-30e* Suppresses DENV Replication by promoting NF-kB–Dependent IFN Production	human monocyte cell line U937

Epigenetic markers associated with host responses to arboviral infections were mostly reported for Dengue [20 studies; (13–16, 18–20, 22–25, 29–31, 34–36, 39–42), followed by Zika [4 studies (33, 38, 41, 43)], Japanese encephalitis [4 studies(26, 28, 32, 44)], Chikungunya [2 studies;(17, 21)], West Nile [1 study (19)], and Congo Crimean [1 study;(37)] viruses. Overall, the studies investigated a number of epigenetic modifications, notably miRNAs/siRNAs [25 studies,(13, 14, 16, 18–20, 22–25, 29–31, 34–36, 39, 40, 42)], iRNA suppression [3 studies (15, 25, 32)], modifications of iRNA regulators [2 studies (19)], and DNA methylation [1 study (13)]. Only a single study described interactions of viral proteins with histones (13).

### Dengue Virus (DENV)

The majority of the studies included in this review investigated DENV, nevertheless these studies analyzed different serotypes and cells types. Overall, 20 studies investigated epigenetic markers associated with DENV infection (Table 1). Fourteen of them conducted *in vitro* studies, using a variety of human cell lines (including macrophages/monocytes, hematopoietic cells, Huh7 hepatocarcinoma cells, EAhy926 endothelial cells, HepG2 liver cancer cells and A-549 lung cancer cells). Six studies investigated markers in the blood of clinical samples. Most studies focused on a specific DENV serotype, i.e., DENV-2 (10 studies), DENV-4 (2 studies), DENV-1 (1 study). The other seven studies did not inform the DENV serotype. A description of the *in vitro* and *in vivo* studies included is summarized separately in the sections below.

#### Epigenetic Mechanisms Associated With DENV Infection Investigated by *in vitro* Studies.

Most DENV *in vitro* studies centered in investigating the interaction of specific DENV serotypes with human cell lines and the elicited epigenetic changes associated with viral replication or the host immune response. These studies focused mainly on iRNA-based mechanisms with eight of them investigating miRNA/siRNA expression. Of these, two used high-throughput tools to determine expression profiles, while the remaining six examined miRNAs associated with DENV infection defined *a priori*. The former assessed profiles following infection of human cell lines with DENV, determining alterations and downstream targets associated with viral replication. Diosa-Toro et al. (39) used small RNA-Seq analysis to investigate the miRNAome and its role in the infection of human macrophages by DENV. They found that only miR-3614-5p was upregulated in DENV negative cells and its overexpression reduced DENV infectivity. By contrast, miR-3614-5p modified the expression of adenosine deaminases acting on RNA (ADAR1), an RNA-editing enzyme that was expected to have an antiviral role but instead it has been reported to facilitate viral replication. Their findings support a role for miR-3614 as a regulator of DENV infection. The other study by Escalera-Cueto et al. (24) used microarray and quantitative PCR to study the expression of miRNAs in human hepatoma cells (Huh-7) infected with DENV-2. Whilst investigating the longitudinal expression profiles of miRNAs, the authors observed that only Let-7c was overexpressed at different time points after infection with DENV-2, with a peak at 12 h post-infection. Let-7c overexpression seemingly correlated with the reduction of DENV replication through the targeting of the transcription factor BACH-1, whose primary downstream target is the HO-1 enzyme, a marker associated with cellular oxidative stress. As such, the downregulation of BACH-1 is implicated with the upregulation of HO-1 and these findings support the fact that Let-7c has an antiviral effect through the modulation of BACH-1 and HO-1, enhancing cellular oxidative stress. Overall, the two studies above provide evidence for epigenetic parameters associated with viral replication, with each supporting specific miRNAs and downstream targets although this may be due to specific events arising from the use of distinct cell lines and/or DENV serotypes.

Wu et al. (22) identified the role of miR-223 in the regulation of DENV-2 replication and then used three software packages (miRanda, PicTar, and Target Scan) to predict the target genes for miR-223. The analysis suggested that miR-223 targeted the microtubule destabilizer *STMN1* gene, and subsequent experiments demonstrated high levels of *STMN1* during viral replication. In short, the study suggests that miR-223 controls DENV-2 replication by negatively regulating *STMN1*. The same group also looked at the role of miR-146a in DENV replication using human monocytic cells infected with DENV-1, 2, or 3 serotypes (18). The expression levels of miR-146a measured by quantitative PCR were significantly increased after the infection by any of the DENV serotypes. These changes correlate with decreased levels of the TNFR-associated factor 6 (TRAF6) protein and secreted interferon (IFN)-β. The study goes on to suggest that miR-146a facilitates DENV replication by targeting TRAF6 and consequently reducing IFN-β levels.

Pham et al. (14) investigated the role of hematopoietic cells in modulating DENV replication by targeting miR-142, one of the most abundant hematopoietic-specific miRNAs. After infection with DENV-2, they showed that the incorporation of miR-142 onto the 3'UTR of gene target sites induced an endogenous attenuation of the virus in hematopoietic but not in non-hematopoietic cells.

A recent work by Kanokudom et al. (40) examined the expression of 7 miRNAs chosen from the miRbase. Following infection of human liver cancer cell line HepG2 with DENV-2, only miR-21 was differently expressed, in which its upregulation was found to promote DENV replication.

Zhu et al. (23) looked at the possible role of miR-30e^*^ in the modulation of innate immunity associated with DENV infection. Human monocyte cell lines were infected with serotypes DENV-1, DENV-2 or DENV-3, and the expression of miR-30e^*^ was measured by quantitative PCR. The results showed that infection with all three serotypes significantly induced miR-30e^*^ expression. Furthermore, silencing miR-30e^*^ was associated with an increase in DENV replication irrespective of the serotype tested. A further investigation into downstream targets showed that miR-30e^*^ significantly induced the expression of IFN-β protein. Finally, the simultaneous overexpression of the protein IκBα ORF (without 3′-UTR) in miR-30e^*^-overexpressed cells restored cellular IκBα protein levels. Altogether, this suggests that miR-30e^*^ directly targets the 3′-UTR sequences of IκBα, thus enhancing IFN-β production and consequently suppressing DENV replication.

Recent work by Pu et al. (42) on the role of miR-146a in the autophagy pathway induced by DENV-2 infection in human lung carcinoma epithelial (A-549) cells confirmed that the overexpression of miR-146a was associated with decreased levels of TRAF6. This protein suppresses autophagossome formation and reduces the microtubule-associated protein light-chain 3 (LC3), thus constituting in a marker for authophagy. These results suggest that miR-146a is a negative regulator of DENV-induced autophagy, and TRAF6 is a key target of this microRNA mediating this process. Altogether, the modulation of DENV-associated targets can potentially suppress the excessive inflammation in host cells, thereby lessening the pathological damage caused by DENV infection.

The host iRNA response is regulated by ribonucleases that control the processing of miRNAs and siRNAs, such as Dicer, Drosha, Ago1, and Ago2 complexes (45). These enzymes could be targeted by viral components to regulate host iRNA mechanisms. Overall, five studies investigated the association of DENV infection and the function of these critical enzymes.

Kakumani et al. (15) demonstrated that the infection of human hepatoma Huh7 cells by DENV reduces the expression of the iRNA response regulators, including Dicer, Drosha, Ago1, and Ago2. Furthermore, they showed that DENV replication increased after the suppression of these iRNA regulators. The authors demonstrated that the non-strutural viral protein 4B (dvNS4B) acts as an iRNA suppressor. In fact, NS4B mediates iRNA suppression via transmembrane domains 3 and 5 (TMD3 and TMD5) irrespective of its interferon antagonistic properties, as NS4B can directly inhibit the dicing process. A follow-up study from the same group (25) showed that the DENV nonstructural protein 3 (dvNS3) is another DENV component that potentially acts as a iRNA suppressor in human cell lines. Conversely, Weiskopt et al. (46) reported that NS3 is the main target of CD8+ T cells response, which play a crucial role in the control of DENV infection, though in the context of antibody-dependent enhancement of infection, this may lead to the occurrence of severe dengue. These findings support a role for dvNS3 in favoring viral replication, either by protecting the viral genome from degradation, or by operating the host transcript levels through miRNA regulation. In summary, these studies suggest that DENV encodes suppressing proteins to regulate its replication in mammalian cell lines. Finally, another follow-up study (31) identified that the mitochondrial heat-shock protein GRP75 (which is also part of the human Dicer complex) is involved in the processing of hsa-miR-126, which restricts DENV replication in human cell lines.

Casseb et al. (29) investigated the expression levels of the genes encoding Drosha, DGCR8, and Dicer after the infection of human A-549 cells with DENV-4. DGCR8 is a double-stranded RNA-binding protein that interacts with Drosha and facilitates miRNA maturation. The mRNA levels of Dicer, Drosha, and DGCR8 were all significantly downregulated following the infection, bottoming out at 3 days post-infection and coinciding with an increase in viral replication. Given these DENV-4-mediated changes mirror events described *in vivo* in a timely manner and implicate the toll-like receptors (TLR) and interleukin (IL-1) signaling pathways, authors concluded that the changes in the gene expression pattern of iRNA regulators were directly influencing the innate immune system of the host.

Bogred et al. (19) employed human cell lines that entirely lacked Dicer function to provide better insights into the effects of miRNAs on DENV replication. The study had two principal rationales. First, if a virus is dependent on a particular miRNA, Dicer-deficient cells should be partly or entirely non-permissive with the virus replication. Similarly, had an endogenous miRNA or siRNA impaired viral replication, then the replication process would be accelerated in Dicer-deficient cells. Conversely, their findings appear to suggest that, in general, viral replication in humans is, neither inhibited, nor enhanced by the complete loss of endogenous human miRNAs, which in turn supports the fact that wild-type viruses have evolved mechanisms to evade host miRNA-mediated suppression of the viral gene expression during the replication cycles. Since DENV replication may not necessarily depend on the presence/absence of endogenous miRNAs, their study inferred that the manipulation of miRNA levels may not be as effective to tackle DENV replication as previously thought.

Lastly, a single study investigated the association of histone function with DENV infection and host response. Using the human liver cell line Huh7 infected with DENV-2 serotype, Colpitts et al. (13) used a tandem-affinity purification (TAP) assay to assess which nuclear proteins of the host cell were bound to the DENV-capsid protein (C). They found that the nuclear histone proteins H2A, H2B, H3, and H4 were in fact specific targets of DENV C. The colocalization of viral proteins with all four histones in the cytoplasm of infected cells demonstrated the formation of dimers, which interestingly were resistant to both heat and denaturation. Therefore, DENV C may either bind to histones before they enter the nucleus, or pull them into the cytoplasm after getting translocated into the nucleus and binding to the histones. In addition, DENV C binds cellular DNA, either directly or by forming a complex with core histones. This leads to disruption of histone dimerization and nucleosome formation, thus resulting in gene expression alterations, impairment of DNA transcription, increased DNA damage and, allegedly, a shift favoring the translation of viral over cellular mRNA. The authors also reported that the levels of the four histone proteins examined increased following the infection and that the presence of the DENV-2 virus was associated with changes in H2A phosphorylation over time. At 24 h post-infection, phosphorylation of H2A was significantly higher than in uninfected cells. In contract, the data revealed decreased phosphorylation at 48 h post-infection, whilst the levels increased again at both 72 and 96 h post-infection. In conclusion, this study suggests that the capsid protein of the DENV-2 virus may target histones to disrupt normal host cell genetic machinery in favor of viral replication and perpetuation of its life cycle.

#### Epigenetic Mechanisms Associated With DENV Infection Investigated by *in vivo* Studies

Several studies investigated blood samples of DENV-infected patients to prospect epigenetic indicators with diagnostic and/or therapeutic potential.

Ouyang et al. (35) collected a total of 72 serum specimens (i.e., 40 patients with active DENV-1 replication and 32 healthy controls). Initially six serum samples (3 patients and 3 controls) were profiled using miRNA PCR arrays in blood. Compared to healthy controls, 41 miRNAs were found to be upregulated and a further 12 miRNAs were downregulated in the sera of DENV-1 patients. The validation analysis was performed with the remaining samples using quantitative PCR and confirmed that only serum miR-21-5p, miR-590-5p, miR-188-5p, and miR-152-3p were upregulated, while only miR-146a-5p was downregulated in DENV-1-infected patients. Receiver operating characteristic (ROC) curve and correlation analyses were performed to evaluate the potential of these miRNAs for the diagnosis of DENV infection. ROC curve suggested that only serum miR-21-5p and miR-146a-5p could reasonably discriminate DENV-infected patients from healthy controls.

Tambyah et al. (36) investigated blood samples of DENV-infected patients to shortlist miRNAs that could be associated with the pathophysiological mechanisms. Patients infected by influenza virus were used as a comparison group to determine the specificity of the differentially expressed miRNAs to DENV infection. Results showed that 12 miRNAs (miR-450b-5p, miR-491-5p, miR-499a-3p, miR-512-5p, miR-615-5p, miR-624-5p, miR-892b, miR-1204, miR-1225-5p, miR-3121-3p, miR-4259, and miR-4327) were specifically linked with acute dengue cases.

Qi et al. (16) reported results from miRNA and inflammatory cytokine profiles from the peripheral blood mononuclear cells (PBMCs) of 24 DENV-2-infected patients. Changes in the expression of cytokines were investigated using multiplex arrays with levels of the chemokine (C-C motif) ligand 5 (CCL5), IL-6, and IL-8 were increased in DENV-2-infected PBMCs in comparison to uninfected PMBCs, while levels of TNF-α, IL-10, MCP-1, and CCL4 were decreased compared to uninfected PBMCs. Their initial miRNA array analysis identified 11 upregulated and 4 downregulated miRNAs with replication analysis using quantitative PCR analyses confirming that miR-106b, miR-20a, and miR-30b were downregulated in DENV-2-infected PMBCs, while miR-4290, miR-let-7e, miR-1290, and miR-33a were found to be upregulated. Whereas follow-up *in silico* analyses suggested that miR-106b may target CCL5 mRNA, miR-let-7e were found to inhibit CCL3 and IL-6 mRNA expression. Altogether, this supports a role for miRNAs to modulate immune activation of DENV-2-infected PMBCs, highlighting that specific miRNAs target specific inflammatory mediators.

Chen et al. (20) investigated the role of miRNAs in the regulation of suppressor of cytokine signaling 1 (SOCS1) and its association with dengue haemorrhagic fever (DHF). *SOCS1* is a negative regulator of cytokine signaling and may be involved in the development of DHF during DENV infection. Quantitative PCR was used to measure *SOCS1* mRNA and its regulatory influence on miRNA levels in PBMCs obtained from the blood of patients with non-hemorrhagic dengue fever (DF) vs DHF. Patients with DF but not DHF had higher levels of *SOCS1*, as well as increased production of IFN-γ. On the other hand, DHF patients had increased production of IL-10. miR-221 and miR-572 levels were higher in patients with DF when compared to DHF. On the other hand, DF patients had decreased levels of miR-150 in comparison to DHF. With respect to *SOCS1* mRNA expression, this was inversely correlated with miR-150 expression in the PMBCs of patients with DHF, with an *in vitro* experiment replicating this association using DENV-2-infected PMBCs. Altogether, these findings support that *SOCS1* expression is reduced in PBMCs derived from patients with DHF, which in turn was significantly associated with the upregulation of miR-150 levels in a dose-dependent manner.

Liu et al. (34) studied the role of miRNAs in the regulation of granzyme B (GrzB), a serine protease found in the granules of cytotoxic lymphocytes (CTLs), natural killer cells (NK cells) and cytotoxic T cells. Software packages miRanda and TargetScan were used to predict sequences of miRNAs that potentially bind to the 3'-UTR regions of perforin (PRF1) and GrzB mRNA sequences, and as a result regulating the expression of these cytotoxic molecules in humans. They measured expression levels of the miRNAs selected by *in silico* analysis (i.e., miR-27a^*^, miR-30e, and miR-378) using quantitative PCR assays. All three miRNAs were reported to be downregulated, but miR-378 exhibited remarkably higher binding potential to the 3'-UTR of GrzB compared to the analogous PRF1 mRNA sequence. Therefore, this study suggests that miR-378 is a key player in the host immune response by modulating NK cell GrzB production during acute DENV infection.

A single study examined the gene methylation status of genes encoding for proteins modulating the innate immune response. Gomes et al. (30) used methylation-specific PCR assays to identify the methylation status of the IFN-γ and TNF-α gene promoters in the DNA extracted from peripheral blood from DENV infected subjects. Their comparative analysis showed that these patients had significant demethylation in the TNF-α but not in the IFN-γ promoter regions.

### Zika Virus (ZIKV)

In total, four *in vitro* studies investigated epigenetic modifications associated with ZIKV infection.

Bayer et al. (43) investigated the hypothesis that trophoblastic chromosome 19 miRNA cluster (C19MC) expressed in primary human trophoblasts (PHTs) might reduce ZIKV infection in non-trophoblastic cells. PHTs are relatively resistant to infection by several viruses. The fact that the resistance to ZIKV is lost in embryonic stem cells when they are differentiated into an early trophoblast lineage suggests that term trophoblasts utilize a diverse array of antiviral pathways to protect the placenta, and consequently, the fetus, from ZIKV infection. However, at very early stages of pregnancy, when embryonic lineages are being differentiated, there is a probable period of trophoblast vulnerability that seemingly ends when the subsequent villous placenta emerges and begins to mature. Therefore, C19MC could regulate the susceptibility of non-trophoblastic cells to ZIKV. In their work, human osteosarcoma U2OS cells were transfected with the most highly expressed members of C19MC (miR-512-3p, miR-516b, miR-517a, and miR-525-5p) and infected with ZIKV. C19MC miRNA reduced ZIKV infection, as denoted by a 50% drop in the production of viral RNA. As PHT cells can also regulate type III IFNs as a secondary pathway to achieve resistance to infection, it was demonstrated that C19MC does not exert any effect on interferon-stimulated genes, and that type III IFNs do not affect the expression levels of miRNAs in U2OS cells. Together, these findings provide evidence that the miRNAs stimulate resistance to the disease through a type III IFN-independent pathway.

Devhare et al. (38) investigated mechanisms associated with ZIKV-mediated impairment of human neural stem cells (hNSC) differentiation and progenitor cell growth, and their relationship with different sets of ZIKV strains. The hNSCs were infected with either the African, the Asian ZIKV strains, or both. Cells infected with both strains displayed distinct E glycoprotein localization and growth pattern. Their results showed an upregulation of several genes upon viral infection and a more effective growth of African ZIKV in hNSCs (though authors mentioned this may be in part due to this variant being utilized for several passages in cell culture), but not of Asian ZIKV, for which less studies were also documented. Furthermore, expression of genes associated with cell cycle arrest were enhanced in virus-infected hNSCs, consequently inducing DNA damage responses though this might be dependent on specific virus strain's mechanisms. They also demonstrated a modest increase in p53 Ser15 phosphorylation, a marker of p53 functional activation, in Asian Zika virus-infected hNSCs. However, African Zika virus-infected cells exhibited reduced p53 phosphorylation. Lastly, it was analyzed the expression levels of p53 targets p21 and PUMA and, interestingly, p21 was detected only in Asian ZIKV-infected hNSCs, potentially promoting cell cycle arrest and the capacity to limit the DNA damage. Conversely, the African strain led to a more apoptotic cell death aspect.

Kozak et al. (41) studied the profile and temporal changes of host miRNAs and transcriptome associated with ZIKV. Human embryonic astrocytes were infected with ZIKV strain PRVABC59, and changes in miRNAs expression was measured 24, 48, or 72 h post-infection using several techniques, including immuno?uorescence, quantitative PCR and microarrays. Their findings suggest that there was a trend toward an overall decrease in miRNA expression in the cells over the course of ZIKV infection. A subset of miRNAs was found to be upregulated during infection, including miR-30e-3p, miR-30e-5p, and miR-17-5p.

Changes in the expression profile of candidate target genes suggest that the unfolded protein response pathway are a major target for ZIKV-mediated regulation, since they observed at 48 h post infection an induction of genetic pathways related to authophagy, including *CHOP* and *GADD34*.

Lichinchi et al. (33) investigated the methylation status of ZIKV RNA and the role of these epigenetic modifications in the host. Authors reported that host methyltransferases and demethylases control N6-Methyladenosine (m^6^A) modification in ZIKV RNA. A metagene analysis of host epigenetic modifications showed that ZIKV infection increased m^6^A levels in the 5′UTR but comparatively decreased in 3'UTR target positions of the human transcriptome. Gene ontology analyses of these genes implicated a number of immune-related mediators when m^6^A levels peak in the host.

### Japanese Encephalitis Virus (JEV)

Two *in vitro* studies used high-throughput screening tools to assess miRNA expression profiles in human microglial cells infected with JEV.

Kumari et al. (32) employed microarrays to investigate levels of miRNAs at 6, 24, or 48 h post-infection, aiming to identify overlapping patterns of differentially expressed markers associated with JEV. Initially only 36% of miRNAs were upregulated at 6 h post-infection, peaking at 24 h with a proportion of 71% of the investigated miRNAs and remaining high at 48 h post-infection (64%). A consistent upregulation across the time series was observed for a specific group of miRNAs, including miR-3648, miR-129-5p, miR-3687, and miR-572. The same study also found downregulation of miRNAs after infection of microglial cells with JEV. At 48 h post infection miR-128, miR-132, miR-222, and miR-130b were found to be downregulated.

Authors also investigated the role of miRNAs in regulating the expression of genes associated with the host immune response. Since activation of Notch pathway during JEV infection is modulated by miR-34c-5p and this probably binds 3′UTR of Notch gene, overexpression of miR-34c-5p is expected to lead to attenuation of JEV induced TNF and IL-6 production by microglia. They observed no apparent impact on the viral replication since no significant changes in jevNS1 protein expression were detected.

Rastogi et al. (44) used microarrays to investigate changes in the expression of 526 miRNA in JEV-infected human microglial cells. They found that 48 miRNAs were differentially expressed: 34 were upregulated, while a further 14 were downregulated. Bioinformatics tools were used to identify the targets of the differentially expressed miRNA. The upregulated miRNAs were involved in pathways associated with several processes, including the maintenance of endothelial barrier and adhesion junctions, ubiquitin-mediated signaling and protein processing in the endoplasmic reticulum, and apoptosis. The downregulated miRNAs were associated with pathways mediating proinflammatory responses, cytokine and chemokine expression and neuropeptide signaling pathway. Although the miRNA-target interactions were not specifically investigated, authors speculate on four possible mechanisms associated with susceptibility of microglial cells to JEV infection that would be related to the changes in miRNA profiles. The miR-32-5p appeared to be upregulated and the Kruppel-like-factor 4 (*KLF4*) gene was predicted as one of its target. This gene has been reported to regulate endothelial barrier function in glioma cells, by suppressing the promoter activity of tight junction proteins. Thus, regulation of the blood-brain barrier might be a pathological mechanism associated with JEV infection. Moreover, miR-29b-3p was found to be upregulated, and Tumor Necrosis Factor receptor associated factor 4 (*TRAF4*) was identified as its downstream target. *TRAF4* regulates cell survival, and since microglial cells are activated but never do they undergo apoptosis during JEV infection, this might be the result of *TRAF4* suppression, which inhibits apoptosis and promotes cell survival. The expression of miR-205-5p was suppressed after JEV infection, and its predicted target was the NFAT5 gene, which is associated with neuroinflammation. Therefore, regulation of this pathway following Japanese encephalitis may result in inflammation and BBB changes. Finally, miR-432-5p was downregulated, and its potential target gene, neural precursor cell expressed developmentally down-regulated protein 4 (*NEDD4L*), is involved in the ubiquitin-mediated proteolysis pathway and has been reported to promote and suppress the rate of virus release from infected cells. Therefore, miR-432-5p may play a critical role in regulating virus release from microglial cells during JEV infection.

Another two *in vitro* studies investigated specific miRNAs associated with JEV infection. Zhu et al. (28) used the human astrocytoma cell line U251 to assess the role of miR-15b as a regulator of JEV-induced inflammation through targeting of ring finger protein 125 (RNF125), which may regulate the T-cell receptor signaling pathway. This study showed that virus-mediated induction of miR-15b increases RIG-I expression by direct suppression of the target gene *RNF125*, resulting in the aggravation of JEV-induced production of inflammatory mediators. miR-15b is also involved in the regulation of chemokine- and cytokine-mediated inflammation pathways, including TNF-α, IL-1β, IL-6, CCL5, IL-12p70, and CCL2. Therefore, authors finally showed that these immunological mediators were significantly upregulated during JEV infection and the inhibition of endogenous miR-15b using antisense miRNA significantly decreased JEV-triggered cytokine production.

Sharma et al. (26) studied the anti-inflammatory role of miR-146a during infection with the JEV strain JaOArS982. They used quantitative PCR and luciferase assays to determine the influence of miR-146a on the *in vitro* expression of interferon-stimulated genes using human microglial cells. Infection with JEV upregulated miR-146a, which was associated with the gene expression downregulation of *TRAF6, IRAK1, IRAK2*, and *STAT1*. The exogenous overexpression of miR-146a suppressed NF-κB activation and decreased the Jak-STAT pathway activity associated with JEV infection, which in turn led to the downregulation of interferon-stimulated genes (*IFIT-1* and *IFIT-2*) and ultimately facilitated viral replication.

### West Nile Virus (WNV)

Two *in vitro* studies investigated the role of epigenetic modifications in the human response to WNV infection. Both studies used high-throughput techniques to investigate iRNA-based mechanisms.

Slonchak et al. (27) studied the role of host miRNA during WNV human infection. The authors employed RNASeq analysis to determine changes in the expression of host miRNAs in human embryonic kidney 293 (HEK293) cell line. In comparison to the uninfected controls, multiple miRNAs were differentially expressed in the infected cell group at 24 and 48 hours post-infection. Only three miRNAs were significantly upregulated: miR-1271-5p, miR-532-5p, and miR-1307-3p. To understand their functions, HEK293 cells were transfected with either miRNA-specific or nonspecific miRNA mimics or inhibitors, and then infected with WNV. WNV titers appeared to be similar to controls whenever the miR-1271-5p and miR-1307-3p mimics or the inhibitors were transfected, thus indicating that these miRNAs did not influence viral replication. In contrast, WNV titers were significantly reduced in cells transfected with the miR-532-5p mimic and increased in cells transfected with the miR-532-5p inhibitor. A genome-wide computational prediction of miR-532-5p targets shortlisted 10 potential targets. In the follow-up investigation, only the for *TAB3* and *SESTD1* mRNAs were reported to be decreased following WNV infection. Altogether, this suggests that the upregulation of miR-532-5p and subsequent suppression of *SESTD1* and *TAB3* genes represent a host antiviral response during WNV infection.

Bogred et al. (19) studied the influence of endogenous miRNAs and virus-induced siRNAs on WNV replication. In a first experiment, human Huh7 cells were infected with WNV and the expression of miRNAs/siRNAs was examined using deep sequencing. They noticed that infected cells did not express detectable levels of miRNAs and/or siRNAs. In a second experiment, authors used a human cell line that did not express Dicer, and, therefore, were unable to produce miRNAs or siRNAs. No significant differences in viral replication were observed in WNV-infected cells in relation to the controls with unchanged Dicer activity. Altogether, the results show that WNV gene expression, similarly to the findings described above for DENV, is not suppressed by endogenous miRNAs which points to the fact that viruses may have evolved mechanisms in an attempt to evade or bypass human miRNA-based defenses.

### Chikungunya Virus (CHIKV)

Only two *in vitro* studies investigated epigenetic modifications associated with CHIKV infection. One used high-throughput tools to screen for potential diagnostic markers or therapeutic targets (17), while the other explicitly investigated miR-146a (21).

The first study described miRNA signatures associated with CHIKV infection from three different human cell lines: human embryonic kidney cell line HEK293T, Vero-76, and primary human dermal fibroblast cells. They employed custom microarrays to identify miRNAs with altered expression patterns. A total of 59 miRNAs were found to be upregulated, while a further 33 were downregulated at 12 and 24 hours post CHIKV infection. Approximately 53 and 45% of upregulated and downregulated miRNAs, respectively, have been implicated with other viral infections, particularly HCV, HBV, HPV and HIV1. These authors also investigated changes in the expression of small nucleolar RNAs (snoRNAs), an epigenetic mechanism that guides chemical modifications of other RNAs, mainly ribosomal RNAs (47). Findings showed that 48 snoRNAs were upregulated. Of these, 16 were of the C/D type, known to be associated to ribosomal RNA acetylation.

Conversely, the second study investigated the role of miR-146a in the regulation of inflammatory responses associated with CHIKV infection in human synovial fibroblasts. CHIKV infection was associated with increased cellular expression of miR-146a and subsequent gene expression downregulation of *TRAF6, IRAK1*, and *IRAK*, as well as increased viral replication. Therefore, They concluded that CHIKV reduced anti-viral immune response in human fibroblasts through enhanced expression of miR-146a.

### Crimean Congo Virus (CCV)

A single *in vivo* study investigated CCV-associated epigenetic factors. Demir et al profiled miRNA expression of plasma samples from eight individuals with confirmed Crimean-Congo hemorrhagic fever (CCHF) diagnosis (37). Their results identified 106 CCV-associated miRNAs that were differentially expressed in peripheral blood mononuclear cells. Main upregulated signatures were miR-144, miR-451, miR-486-5p, miR-608, miR-363, miR-31, miR-32, miR-575, miR-632, and -miR-541 in CCHF patients. Out of that, several targets were implicated with the immune cell activation, cellular adhesion and signal transduction. Authors highlighted that a similar cytokine imbalance occurs during viral hemorrhagic fevers, regardless of the etiolopathological agent, and that could be the result of disease processes mediated by shared epigenetic alterations.

Furthermore, chief downregulated miRNA leads were miR-493, miR-889, miR-655, miR-656, miR-26a-1, miR-154, miR-335, miR-1197, and miR-146a. They proposed that at least some of these candidates were related to proinflammatory cytokine-induced alterations on the endothelium, leading to vascular dysfunctions and the development of hemorrhagic damage seen in CCHF patients.

Altogether, their findings suggest a key role for miRNAs in regulating human immune response to CCV and modulating disease progress and CCHF clinical manifestations.

## Discussion

In general terms there are five well-characterized processes that appear to mediate epigenetic regulation, namely DNA methylation, nucleosome positioning, histone variants, histone modifications, and regulatory RNA class family. While there is a large body of evidence suggesting that epigenetic factors play a critical role in the regulation of virus infections, in many cases the detailed mechanisms have not been elucidated. Although several limiting factors contribute to our relative lack of understanding of the intricacies of viral epigenetic regulation, the inherent complexity of epigenetic regulation most likely constitutes in the single chief hurdle. To our knowledge, this is the first review that aimed to synthetize epigenetic changes associated with arboviruses infections in the human host.

Our results reveal a significant heterogeneity across studies, with authors frequently exploring varied hypotheses and distinct sets of markers, which unquestionably restricted our abilities to draw direct comparisons and undertake a substantiated meta-analysis. Our review showed that the most frequent epigenetic events expressed during arboviruses infections were related to modifications in miRNA profiles with a number of leads implicated. Aside of this, alterations in the dicer complex and dicer-like enzymes were also reported by two independent studies (19, 29). Only a single study found association with histone modifications (13) with a further investigation describing changes in the DNA methylation pattern (30). Another study reported alterations in p53 phosphorylation using an infection model of ZIKV (38).

The most frequent human miRNA alteration implicated with arbovirus infections was the miR-146. This miRNA may constitute in an interesting biomarker as it was upregulated during infections with JEV, CHKV and DENV (18, 21, 26) though it was downregulated during CCHF (37). Two of the revised studies found miR-146 levels to be in perceivable downregulation following CCHF infection (37), suggesting that its reduction following infection led to the secretion of proinflammatory mediators. Of note, CCHF is the only non-mosquito-borne arbovirus included in this review but comes from a family almost exclusive of tick-borne viruses (*Nairoviridae*), and given these particularities will most likely elicit a rather distinct pathophysiological response compared to those of mosquito-borne viruses. Irrespective of that, not all findings were consistent since a recent study found that miR-146 was downregulated during DENV infection (35). Further to this, miR-146 seems to be a key inflammatory mediator that stimulates the release of cytokines during innate immune response (48), however it seems to work through a divisive role as a proviral factor as it targets IRAK1 and TRAF6 expression leading to the inhibition of IFN production, thus hindering antiviral host defenses (49). It is possible that its upregulation observed during infections with JEV, CHKV and DENV may actually the result of increased host susceptibility to these diseases, therefore warranting further investigations to determine its clinical utility. The miR-146 has also been proposed to be a marker of sepsis, as well as a target to reduce immune hyperactivation during infection (50).

Our review also supports the involvement of other host miRNA markers as epigenetic modulators of arbovirus infections. For instance, miR-30e has been linked with NF-kB activation and IFN-β production, and its altered levels generally associated with restriction of DENV infection, via positively modulating the antiviral innate immune response (23, 51). While another study (41) showed that miR-30e is involved in an autophagic pathway in patients infected with ZIKV, others proposed that miR-30e had no actual role in regulating expression of granzyme B in NK cells during DENV infection (34), an important cytotoxic molecule to control viral replication. Despite a growing body of evidence for miR-30e, a consensus on its actual role in modulating the human immune system following viral infection has yet to be reached. Similarly, Dicer, a class III endoribonuclease, cleaves dsRNA into siRNA and miRNA. Both siRNA and miRNA facilitate the activation of RNA-induced silencing complex (RISC), which is essential for RNA interference characteristics (9). In other words, since both processing enzymes Drosh and Dice, process the majority of pri-miRNA/pre-miRNAs into duplex mature miRNAs, which is essential for rendering their biological properties, alterations in their levels as a result of an infection would not be confined to arbovirus and would not constitute in a specific molecular signature. Indeed, another review indicated that cellular miRNAs physiologically regulate the replication of other mammalian viruses with deliberate repression of mammalian Dicer increasing the replication of HIV-1, vesicular stomatitis virus (VSV) and influenza A virus (52).

All in all, our review suggests that epigenetic mechanisms accompanying arbovirus infections may be rather specific and distinct to the ones associated with other common viral diseases, such as DNA tumor viruses where alterations in miRNA expression patterns are not so frequent (10). These viruses have predominantly been associated with DNA methylation events in the promoter positions of key disease-associated genes (10). Similarly, DNA methylation is part of the typical gene silencing process that occurs during the latent infection by the herpes simplex virus (HSV) family (10). Conversely, polyomavirus and adenovirus frequently have their viral genome integrated into the host genome with some studies reporting these viruses may also employ DNA methylation mechanisms during the course of the infection (10). A widely described virus-driven epigenetic modification is the one associated with Papilomavirus that implicates histone modifications (10).Comparatively, Hepatitis B virus possesses a mini chromosome with nucleosome and histone modifications, which appears to dysregulate a number of cellular pathways, in part by binding to genomic DNA, by changing expression patterns of miRNAs, or by connecting to sirtuin1 gene (SIRT1), an intracellular regulatory protein known to regulate epigenetic gene silencing and suppress recombination of rDNA. Overall, this supports that viruses may use different epigenetic mechanisms, and that miRNA may be functionally more relevant for arbovirus infections in humans. Despite that, the significance of miRNAs for the development and progression of arboviral infections and for the host disease response remain largely unclear.

Only a single study reported specific epigenetic alterations when stratifying patients for cardinal clinical features (36). The authors identified differentially expressed miRNAs in patients with mild vs. severe dengue, with which they supported the existence of a relevant epigenetic basis for disease progression and complications seen in some patients. Yet, a major limitation for producing sturdier assertions from the existing literature is the limited number of *in vivo* studies available. Only six studies were encountered and met inclusion criteria of our review, with five of them restricting research to DENV infection only. In these studies, authors were mainly engaged in either, identifying a pattern of epigenetic signatures during the infection (16, 35), or searching evidence for the activation of genes that encode cytokines and the innate immune system, such as TNF-alpha (30). It has been documented that the miRNAs associated with cytokine activation may lead to either side of the disease spectra depending on the pattern of their gene targets and downstream pathways. It has been shown that sustained levels of IFNs, whether or not miRNA-dependent, in DENV-infected humans correlate with protection and sub-clinical disease (53, 54), and their secretion correlates with the induction of a cytotoxic T-cell response (46). For instance, infection with DENV up-regulated the expression of miR-30e^*^, which a play a role controlling the infection through the expression of IFN-β. Conversely, other authors suggest that miR-146a facilitates DENV replication (18) by reducing IFN-β levels, the opposite of what is achieved with the introduction of miR-30a, which highlight the need for carrying out further evidence-based on this topic to support these views.

A clinically relevant question that our review was unable to address concerns the persistence of early response markers, such as IgM anti-Chikungunya antibodies, in chronic patients. These have been documented even after several months since the disease onset, suggesting that single-stranded arbovirus infections are seldom self-resolving processes and certainly not short-lived as many previously speculated (55, 56). Another important limitation of our review is the lack of meaningful literature in relation to specific epigenetic events and lead biomarkers with potential to discriminate the myriad of infections caused by circulating arbovirus worldwide. In Brazil, for instance, over 210 arbovirus species have already been isolated, the vast majority from the Amazon rain forest, with at least 110 new to scientist and 34 proven to cause human infections. (57). Furthermore, arbovirus are RNA entities, very mutagenic and capable of causing renewing public health threats in the form of outbreaks and pandemics. In addition to the highly publicized emergence of ZIKV and CHKV, the last decade has witnessed an expansion of previously localized endemic arbovirus, such as Oropouche (OROV), Mayaro (MAYV), Rocio (RCV), Saint Louis encephalitis (SLEV), Ross River (RRV), and both Eastern, and Western Equine Encephalitis (EEEV and WEEV) viruses from the *Flaviviridae, Togaviridae* and *Bunyaviridae* families, with each almost certainly resulting in specific molecular signatures and distinct patterns of host responses.

In general, arboviruses are zoonosis-causing agents as they are mostly kept in nature by both a non-human vertebrate and a hematophagous arthropod vector cycles. To further compound this, the saliva from blood-feeding arthropods, such as mosquitoes is enriched with active molecules that display a variety of functions leading to the modulation of the host immune system, ultimately facilitating arbovirus evasion and the establishment of the disease (58). Depending on the vector in question and intrinsic human factors, that *per se* can result in specific host-mediated immune and epigenetic responses, adding further complexity to effectively integrating *in vitro* and *in vivo* molecular analyses.

Clearly, arboviruses infections account for a massive disease burden to healthcare systems and to labor productivity, especially in tropical and developing countries (59). This systematic review shows that the major epigenetic alterations associated with arboviruses infections were microRNAs, particularly miR-146 and miR-30e, and the dicer complex. Studies fitting our inclusion criteria have been highly heterogeneous in nature, presenting varied methodological designs and testing for distinct sets of hypotheses. Further research is certainly necessary to appreciate how epigenetic findings could be validated as biomarker tools for diagnosis, staging, and prognosis of arbovirus diseases in humans. Concerted work into the mechanisms of specific epigenetic regulations is likely to support the development of bespoken prevention or therapeutic strategies for arboviral infections.

Certainly, more thorough out epigenetic studies on the interactions of arboviral infections with common chronic diseases in humans, as well as the role of co-infections, would be extremely opportune for meaningful progress in this field. For instance, a major outbreak driven by emerging CHKV variants in Brazil between 2016 and 2017, has reminded scientists and public health authorities of the devastating impacts of arboviral infections and disease chronification on labor productivity and outcomes of underlying diseases, especially diabetes type II (59). The untapped potential of epigenetic markers may constitute the missing link to bolster a deeper understanding of pathogenesis and shared response mechanisms across disease-causing arbovirus species and between strains/serotypes of the same species. Combined with the realization of inter- individual differences in disease susceptibility, the development of effective molecular-based tools, including epigenetics, can deliver critical insights into the dynamics of arboviruses epidemics and recurrent outbreaks, potentially enabling both patient and treatment stratifications and the refinement of effective epidemiological surveillance strategies.

## Author Contributions

GA, CL, SV, DM, and FM designed the study protocol. GA and BD performed the literature search. GA, BD, CL, and FM identified studies for eligibility. BD and GA extracted the data. CL, GA, BD, LC, SV, DM, and FM outlined results and work execution. GA, CL, and FM drafted the initial manuscript. LC, MM, RM, SV, DM, AV, and FM critically reviewed and revised the manuscript. All authors read and approved the final manuscript as submitted.

### Conflict of Interest Statement

The authors declare that the research was conducted in the absence of any commercial or financial relationships that could be construed as a potential conflict of interest.

## Abbreviations

mRNA: messenger RNA (mRNA)
RNAi: RNA interference mechanisms
miRNA: micro RNA
miR: micro RNA marker
siRNA: small interfering RNA
DENV: dengue vírus
ADAR1: Adenosine deaminases acting on RNA
TRAF6: TNFR-associated factor 6
IFN: Interferon
LC3: microtubule-associated protein light-chain 3
TMDs: transmembrane domains
dvNS3: dengue virus non-structural protein 3
TLR: toll-like receptors
IL: interleukin
PBMCs: peripheral blood mononuclear cells
CCL5: chemokine (C-C motif) ligand 5
DHF: dengue haemorrhagic fever
SOCS1: regulation of suppressor of cytokine signaling 1 (SOCS1)
GrzB: granzyme B
ZIKV: Zika virus
NK: natural killer
hNSC: human neural stem cells
JEV: Japanese encephalitis virus
KLF4: Kruppel-like-factor 4
RNF: ring finger protein 125
WNV: West Nile virus
CHIKV: Chikungunya virus
snoRNAs: small nucleolar RNAs
CCV: Crimean Congo virus
CCHF: Crimean-Congo hemorrhagic fever.

## References

[1] Gould E Pettersson J Higgs S Charrel R de Lamballerie X. Emerging arboviruses: why today? One Health. (2017) 4:1–13. 10.1016/j.onehlt.2017.06.001 28785601 PMC5501887

[2] Weaver SC Reisen WK. Present and future arboviral threats. Antiviral Res. (2010) 85:328–45. 10.1016/j.antiviral.2009.10.008 19857523 PMC2815176

[3] Mayer SV Tesh RB Vasilakis N. The emergence of arthropod-borne viral diseases: a global prospective on dengue, chikungunya and zika fevers. Acta Trop. (2017) 166:155–63. 10.1016/j.actatropica.2016.11.020 27876643 PMC5203945

[4] Charrel RN de Lamballerie X Raoult D. Chikungunya outbreaks–the globalization of vectorborne diseases. N Engl J Med. (2007) 356:769–71. 10.1056/NEJMp078013 17314335

[5] Bhatt S Gething PW Brady OJ Messina JP Farlow AW Moyes CL. The global distribution and burden of dengue. Nature. (2013) 496:504–7. 10.1038/nature12060 23563266 PMC3651993

[6] Baud D Gubler DJ Schaub B Lanteri MC Musso D. An update on Zika virus infection. Lancet. (2017) 390:2099–109. 10.1016/S0140-6736(17)31450-2 28647173

[7] Meltzer E. Arboviruses and viral hemorrhagic fevers (VHF). Infect Dis Clin North Am. (2012) 26:479–96. 10.1016/j.idc.2012.02.003 22632650

[8] Coffey LL Vasilakis N Brault AC Powers AM Tripet F Weaver SC. Arbovirus evolution in vivo is constrained by host alternation. PNAS. (2008) 105:6970–5. 10.1073/pnas.0712130105 18458341 PMC2383930

[9] Balakrishnan L Milavetz B. Epigenetic regulation of viral biological processes. Viruses. (2017) 9:e346. 10.3390/v9110346 29149060 PMC5707553

[10] Milavetz BI Balakrishnan L. Viral epigenetics. Methods Mol Biol. (2015) 1238:569–96. 10.1007/978-1-4939-1804-1_30 25421681 PMC4478594

[11] Olson KE Blair CD. Arbovirus-mosquito interactions: RNAi pathway. Curr Opin Virol. (2015) 15:119–26. 10.1016/j.coviro.2015.10.001 26629932 PMC4765169

[12] Moher D Liberati A Tetzlaff J Altman DG. Preferred reporting items for systematic reviews and meta-analyses: the PRISMA statement. J Clin Epidemiol. (2009) 62:1006–12. 10.1016/j.jclinepi.2009.06.005 19631508

[13] Colpitts TM Barthel S Wang P Fikrig E. Dengue virus capsid protein binds core histones and inhibits nucleosome formation in human liver cells. PLoS ONE. (2011) 6:e24365. 10.1371/journal.pone.0024365 21909430 PMC3164731

[14] Pham AM Langlois RA TenOever BR. Replication in cells of hematopoietic origin is necessary for Dengue virus dissemination. PLoS Pathog. (2012) 8:e1002465. 10.1371/journal.ppat.1002465 22241991 PMC3252368

[15] Kakumani PK Ponia SS S RK Sood V Chinnappan M Banerjea AC. Role of RNA interference (RNAi) in dengue virus replication and identification of NS4B as an RNAi suppressor. J. Virol. (2013) 87:8870–83. 10.1128/JVI.02774-12 23741001 PMC3754049

[16] Qi Y Li Y Zhang L Huang J. microRNA expression profiling and bioinformatic analysis of dengue virusinfected peripheral blood mononuclear cells. Mol Med Rep. (2013) 7:791–8. 10.3892/mmr.2013.1288 23354650

[17] Saxena T Tandon B Sharma S Chameettachal S Ray P Ray AR. Combined miRNA and mRNA signature identifies key molecular players and pathways involved in chikungunya virus infection in human cells. PLoS ONE. (2013) 8:e79886. 10.1371/journal.pone.0079886 24278205 PMC3836776

[18] Wu S He L Li Y Wang T Feng L Jiang L. miR-146a facilitates replication of dengue virus by dampening interferon induction by targeting TRAF6. J Infect. (2013) 67:329–41. 10.1016/j.jinf.2013.05.003 23685241

[19] Bogerd HP Skalsky RL Kennedy EM Furuse Y Whisnant AW Flores O. Replication of many human viruses is refractory to inhibition by endogenous cellular microRNAs. J Virol. (2014) 88:8065–76. 10.1128/JVI.00985-14 24807715 PMC4097787

[20] Chen RF Yang KD Lee IK Liu JW Huang CH Lin CY. Augmented miR-150 expression associated with depressed SOCS1 expression involved in dengue haemorrhagic fever. J Infect. (2014) 69:366–74. 10.1016/j.jinf.2014.05.013 24907421

[21] Selvamani SP Mishra R Singh SK. Chikungunya virus exploits miR-146a to regulate NF-kappaB pathway in human synovial fibroblasts. PLoS ONE. (2014) 9:e103624. 10.1371/journal.pone.0103624 25083878 PMC4118904

[22] Wu N Gao N Fan D Wei J Zhang J An J. miR-223 inhibits dengue virus replication by negatively regulating the microtubule-destabilizing protein STMN1 in EAhy926 cells. Microbes Infect. (2014) 16:911–22. 10.1016/j.micinf.2014.08.011 25181337 PMC7110837

[23] Zhu X He Z Hu Y Wen W Lin C Yu J. MicroRNA-30e^*^ suppresses dengue virus replication by promoting NF-kappaB-dependent IFN production. PLoS Negl Trop Dis. (2014) 8:e3088. 10.1371/journal.pntd.0003088 25122182 PMC4133224

[24] Escalera-Cueto M Medina-Martinez I del Angel RM Berumen-Campos J Gutierrez-Escolano AL Yocupicio-Monroy M. Let-7c overexpression inhibits dengue virus replication in human hepatoma Huh-7 cells. Virus Res. (2015) 196:105–12. 10.1016/j.virusres.2014.11.010 25445350

[25] Kakumani PK Rajgokul KS Ponia SS Kaur I Mahanty S Medigeshi GR. Dengue NS3, an RNAi suppressor, modulates the human miRNA pathways through its interacting partner. Biochem J. (2015) 471:89–99. 10.1042/BJ20150445 26221025

[26] Sharma N Verma R Kumawat KL Basu A Singh SK. miR-146a suppresses cellular immune response during Japanese encephalitis virus JaOArS982 strain infection in human microglial cells. J Neuroinflamm. (2015) 12:30. 10.1186/s12974-015-0249-0 25889446 PMC4355369

[27] Slonchak A Shannon RP Pali G Khromykh AA. Human MicroRNA miR-532-5p exhibits antiviral activity against west nile virus via suppression of host genes SESTD1 and TAB3 required for virus replication. J Virol. (2015) 90:2388–402. 10.1128/JVI.02608-15 26676784 PMC4810706

[28] Zhu B Ye J Nie Y Ashraf U Zohaib A Duan X. MicroRNA-15b modulates Japanese encephalitis virus-mediated inflammation via targeting RNF125. J Immunol. (2015) 195:2251–62. 10.4049/jimmunol.1500370 26202983

[29] Casseb SM Simith DB Melo KF Mendonca MH Santos AC Carvalho VL. Drosha, DGCR8, and Dicer mRNAs are down-regulated in human cells infected with dengue virus 4, and play a role in viral pathogenesis. Genet. Mol. Res. (2016) 15:gmr7891. 10.4238/gmr.15027891 27173348

[30] Gomes AV de Souza Morais SM Menezes-Filho SL de Almeida LG Rocha RP Ferreira JM. Demethylation profile of the TNF-alpha promoter gene is associated with high expression of this cytokine in Dengue virus patients. J Med Virol. (2016) 88:1297–302. 10.1002/jmv.24478 26792115

[31] Kakumani PK Medigeshi GR Kaur I Malhotra P Mukherjee SK Bhatnagar RK. Role of human GRP75 in miRNA mediated regulation of dengue virus replication. Gene. (2016) 586:7–11. 10.1016/j.gene.2016.03.053 27039024

[32] Kumari B Jain P Das S Ghosal S Hazra B Trivedi AC. Dynamic changes in global microRNAome and transcriptome reveal complex miRNA-mRNA regulated host response to Japanese encephalitis virus in microglial cells. Sci Rep. (2016) 6:20263. 10.1038/srep20263 26838068 PMC4738309

[33] Lichinchi G Zhao BS Wu Y Lu Z Qin Y He C. Dynamics of human and viral RNA methylation during Zika virus infection. Cell Host Microbe. (2016) 20:666–73. 10.1016/j.chom.2016.10.002 27773536 PMC5155635

[34] Liu S Chen L Zeng Y Si L Guo X Zhou J. Suppressed expression of miR-378 targeting gzmb in NK cells is required to control dengue virus infection. Cell Mol Immunol. (2016) 13:700–8. 10.1038/cmi.2015.52 26166761 PMC5037283

[35] Ouyang X Jiang X Gu D Zhang Y Kong SK Jiang C. Dysregulated serum MiRNA profile and promising biomarkers in dengue-infected patients. Int J Med Sci. (2016) 13:195–205. 10.7150/ijms.13996 26941580 PMC4773284

[36] Tambyah PA Ching CS Sepramaniam S Ali JM Armugam A Jeyaseelan K. microRNA expression in blood of dengue patients. Ann Clin Biochem. (2016) 53(Pt 4):466–76. 10.1177/0004563215604001 26290515

[37] Demir ZC Bastug A Bodur H Ergunay K Ozkul A. MicroRNA expression profiles in patients with acute Crimean Congo hemorrhagic fever reveal possible adjustments to cellular pathways. J Med Virol. (2017) 89:417–22. 10.1002/jmv.24667 27551771

[38] Devhare P Meyer K Steele R Ray RB Ray R. Zika virus infection dysregulates human neural stem cell growth and inhibits differentiation into neuroprogenitor cells. Cell Death Dis. (2017) 8:e3106. 10.1038/cddis.2017.517 29022904 PMC5682681

[39] Diosa-Toro M Echavarria-Consuegra L Flipse J Fernandez GJ Kluiver J van den Berg A. MicroRNA profiling of human primary macrophages exposed to dengue virus identifies miRNA-3614-5p as antiviral and regulator of ADAR1 expression. PLoS Negl Trop Dis. (2017) 11:e0005981. 10.1371/journal.pntd.0005981 29045406 PMC5662241

[40] Kanokudom S Vilaivan T Wikan N Thepparit C Smith DR Assavalapsakul W. miR-21 promotes dengue virus serotype 2 replication in HepG2 cells. Antiviral Res. (2017) 142:169–77. 10.1016/j.antiviral.2017.03.020 28365456

[41] Kozak RA Majer A Biondi MJ Medina SJ Goneau LW Sajesh BV. MicroRNA and mRNA dysregulation in astrocytes infected with Zika virus. Viruses. (2017) 9:e297. 10.3390/v9100297 29036922 PMC5691648

[42] Pu J Wu S Xie H Li Y Yang Z Wu X. miR-146a inhibits dengue-virus-induced autophagy by targeting TRAF6. Arch Virol. (2017) 162:3645–59. 10.1007/s00705-017-3516-9 28825144 PMC7086938

[43] Bayer A Lennemann NJ Ouyang Y Sadovsky E Sheridan MA Roberts RM. Chromosome 19 microRNAs exert antiviral activity independent from type III interferon signaling. Placenta. (2018) 61:33–8. 10.1016/j.placenta.2017.11.004 29277269 PMC5745809

[44] Rastogi M Srivastava N Singh SK. Exploitation of microRNAs by Japanese Encephalitis virus in human microglial cells. J Med Virol. (2018) 90:648–54. 10.1002/jmv.24995 29149532

[45] Agrawal N Dasaradhi PV Mohmmed A Malhotra P Bhatnagar RK Mukherjee SK. RNA interference: biology, mechanism, and applications. Microbiol Mol Biol Rev. (2003) 67:657–85. 10.1128/MMBR.67.4.657-685.2003 14665679 PMC309050

[46] Weiskopf D Angelo MA de Azeredo EL Sidney J Greenbaum JA Fernando AN. Comprehensive analysis of dengue virus-specific responses supports an HLA-linked protective role for CD8+ T cells. Proc Natl Acad Sci USA. (2013) 110:E2046–53. 10.1073/pnas.1305227110 23580623 PMC3670335

[47] Bachellerie JP Cavaille J Huttenhofer A. The expanding snoRNA world. Biochimie. (2002) 84:775–90. 10.1016/S0300-9084(02)01402-5 12457565

[48] Corridoni D Chapman T Ambrose T Simmons A. Emerging mechanisms of innate immunity and their translational potential in inflammatory bowel disease. Front Med (Lausanne). (2018) 5:32. 10.3389/fmed.2018.00032 29515999 PMC5825991

[49] Bruscella P Bottini S Baudesson C Pawlotsky JM Feray C Trabucchi M. Viruses and miRNAs: more friends than foes. Front Microbiol. (2017) 8:824. 10.3389/fmicb.2017.00824 28555130 PMC5430039

[50] Saba R Sorensen DL Booth SA. MicroRNA-146a: a dominant, negative regulator of the innate immune response. Front Immunol. (2014) 5:578. 10.3389/fimmu.2014.00578 25484882 PMC4240164

[51] Jiang L Lin C Song L Wu J Chen B Ying Z. MicroRNA-30e^*^ promotes human glioma cell invasiveness in an orthotopic xenotransplantation model by disrupting the NF-kappaB/IkappaBalpha negative feedback loop. J Clin Invest. (2012) 122:33–47. 10.1172/JCI58849 22156201 PMC3248293

[52] Grassmann R Jeang KT. The roles of microRNAs in mammalian virus infection. Biochim Biophys Acta. (2008) 1779:706–11. 10.1016/j.bbagrm.2008.05.005 18549828 PMC2641032

[53] Gunther VJ Putnak R Eckels KH Mammen MP Scherer JM Lyons A. A human challenge model for dengue infection reveals a possible protective role for sustained interferon gamma levels during the acute phase of illness. Vaccine. (2011) 29:3895–904. 10.1016/j.vaccine.2011.03.038 21443963

[54] Jeewandara C Adikari TN Gomes L Fernando S Fernando RH Perera MK. Functionality of dengue virus specific memory T cell responses in individuals who were hospitalized or who had mild or subclinical dengue infection. PLoS Negl Trop Dis. (2015) 9:e0003673 10.1371/journal.pntd.0003673 25875020 PMC4395258

[55] Schilte C Staikowsky F Couderc T Madec Y Carpentier F Kassab S. Chikungunya virus-associated long-term arthralgia: a 36-month prospective longitudinal study. PLoS Negl Trop Dis. (2013) 7:e2137. 10.1371/journal.pntd.0002137 23556021 PMC3605278

[56] Pierro A Rossini G Gaibani P Finarelli AC Moro ML Landini MP. Persistence of anti-chikungunya virus-specific antibodies in a cohort of patients followed from the acute phase of infection after the 2007 outbreak in Italy. New Microbes New Infect. (2015) 7:23–5. 10.1016/j.nmni.2015.04.002 26106482 PMC4475829

[57] do Rosário Casseb A Casseb LMN da Silva SP da Costa Vasconcelos PF Arbovírus: importante zoonose na Amazônia brasileira. Veterinária e Zootecnia. (2013) 20:391–403.

[58] Andrade BB Teixeira CR Barral A Barral-Netto M. Haematophagous arthropod saliva and host defense system: a tale of tear and blood. An Acad Bras Cienc. (2005) 77:665–93. 10.1590/S0001-37652005000400008 16341443

[59] Cavalcanti LPG D'Angelo SM Lemos DRQ Barreto FKA Siqueira AM Miyajima F. Is the recent increment in attributable deaths to type-2 diabetes (T2D) associated with the latest chikungunya outbreak in a major epidemic area in Brazil? Rev Soc Bras Med Trop. (2018) 51:63–5. 10.1590/0037-8682-0440-2017 29513844

