# Supplementary material for: Evidence for Host Epigenetic Signatures Arising From Arbovirus Infections: A Systematic Review
Source: Front Immunol. 2019 May 31;10:1207. doi: 10.3389/fimmu.2019.01207 (PMC6554415; doi:10.3389/fimmu.2019.01207)
Supplement: Supplementary file 1 [file Table_1.pdf]

Author	Year	Reasons for exclusion
Avilla-Bonilla	2017	not human cells
Castilo	2016	not human cells
Castrillon	2017	not human cells
Chang	2016	not human cells
Chen	2016	not human cells
McLean	2017	in silico study
Pylro	2016	in silico study
Sharma	2015	not human cells
Shind	2015	in silico study
Thounaujon	2014	not human cells
Thounaujon	2014	not human cells
